# Comparing Tuberculosis Diagnostic Yield in Smear/Culture and Xpert^®^ MTB/RIF-Based Algorithms Using a Non-Randomised Stepped-Wedge Design

**DOI:** 10.1371/journal.pone.0150487

**Published:** 2016-03-01

**Authors:** Pren Naidoo, Rory Dunbar, Carl Lombard, Elizabeth du Toit, Judy Caldwell, Anne Detjen, S. Bertel Squire, Donald A. Enarson, Nulda Beyers

**Affiliations:** 1 Desmond Tutu TB Centre, Department of Paediatrics and Child Health, Faculty of Medicine and Health Sciences, Stellenbosch University, Cape Town, South Africa; 2 Biostatistics Unit, South African Medical Research Council, Cape Town, South Africa; 3 City of Cape Town Health Directorate, Cape Town, South Africa; 4 The International Union against TB and Lung Disease, Paris, France; 5 Liverpool School of Tropical Medicine, Liverpool, United Kingdom; University of Malaya, MALAYSIA

## Abstract

**Setting:**

Primary health services in Cape Town, South Africa.

**Study Aim:**

To compare tuberculosis (TB) diagnostic yield in an existing smear/culture-based and a newly introduced Xpert^®^ MTB/RIF-based algorithm.

**Methods:**

TB diagnostic yield (the proportion of presumptive TB cases with a laboratory diagnosis of TB) was assessed using a non-randomised stepped-wedge design as sites transitioned to the Xpert^®^ based algorithm. We identified the full sequence of sputum tests recorded in the electronic laboratory database for presumptive TB cases from 60 primary health sites during seven one-month time-points, six months apart. Differences in TB yield and temporal trends were estimated using a binomial regression model.

**Results:**

TB yield was 20.9% (95% CI 19.9% to 22.0%) in the smear/culture-based algorithm compared to 17.9% (95%CI 16.4% to 19.5%) in the Xpert^®^ based algorithm. There was a decline in TB yield over time with a mean risk difference of -0.9% (95% CI -1.2% to -0.6%) (p<0.001) per time-point. When estimates were adjusted for the temporal trend, TB yield was 19.1% (95% CI 17.6% to 20.5%) in the smear/culture-based algorithm compared to 19.3% (95% CI 17.7% to 20.9%) in the Xpert^®^ based algorithm with a risk difference of 0.3% (95% CI -1.8% to 2.3%) (p = 0.796). Culture tests were undertaken for 35.5% of smear-negative compared to 17.9% of Xpert^®^ negative low MDR-TB risk cases and for 82.6% of smear-negative compared to 40.5% of Xpert^®^ negative high MDR-TB risk cases in respective algorithms.

**Conclusion:**

Introduction of an Xpert^®^ based algorithm did not produce the expected increase in TB diagnostic yield. Studies are required to assess whether improving adherence to the Xpert^®^ negative algorithm for HIV-infected individuals will increase yield. In light of the high cost of Xpert^®^, a review of its role as a screening test for all presumptive TB cases may be warranted.

## Introduction

The World Health Organisation identified five priorities to accelerate progress towards achieving the 2015 United Nation’s Millennium Development Goals for tuberculosis (TB) [1]. The first was to “reach the missed cases”, the estimated 2.9 million TB cases that were not diagnosed or reported in national notification systems. Expanding diagnostic services, including access to rapid tests, was identified as one of the strategies to achieve this.

South Africa, which ranks second on the list of 12 countries contributing 75% of missed cases [1] introduced Xpert^®^ MTB/RIF (Cepheid, Sunnyvale, CA, USA) (Xpert) as a replacement for smear microscopy for all presumptive TB cases in 2011. Xpert is recommended as the initial diagnostic test in individuals suspected of multidrug-resistant (MDR)-TB or human immunodeficiency virus (HIV)-associated TB [2], both of which are prevalent in South Africa. Full Xpert coverage was predicted to identify 30% more TB cases in South Africa in 2013 compared to smear and culture [3].

Xpert has higher sensitivity than smear microscopy. A Cochrane Review of fifteen studies with Xpert replacing smear microscopy as the initial test, yielded a pooled sensitivity of 88% (95%CrI: 83% to 92%) and specificity of 98% (95% CrI: 97% to 99%) for detecting *Mycobacterium tuberculosis* (MTB) [4]. In comparison conventional light microscopy has an average sensitivity of 53.8% for a single smear and 64.9% for two smears [5] with a 10% increase from fluorescence microscopy [6]. Despite the advantages of light microscopy, including simplicity, low cost, high specificity in TB endemic areas and the ability to identify the most infectious cases of TB, its major limitation is low and variable sensitivity, with particularly low sensitivity in HIV prevalent areas [7–9].

Sputum culture, considered the gold standard in TB diagnosis and important in the diagnosis of smear-negative TB, is able to diagnose paucibacillary disease at concentrations as low as 10 bacteria per ml [8] compared to 10,000 bacteria per ml for conventional light microscopy. However, culture is slow, taking 6–8 weeks on solid Löwenstein-Jensen medium and 8–16 days with Mycobacterial Growth Inhibitor Tube (MGIT) liquid culture [7]. Culture is also more expensive than smear microscopy [10,11] and requires sophisticated laboratory infrastructure and technical expertise.

Whilst rapid, more sensitive molecular diagnostic tests such as Xpert have the technical capacity to address the limitations of smear and culture, very little has been reported on their use in routine operational conditions, particularly within diagnostic algorithms. Increased test sensitivity may not on its own translate into an increase in the proportion of bacteriologically confirmed TB cases. The role of the test within the algorithm, how effectively it is used, the use of follow-on tests such as culture and the number of presumptive TB cases screened may all play a role.

The aim of this study was to compare the proportion of presumptive TB cases with a laboratory diagnosis of TB (TB diagnostic yield) in an existing smear/culture-based algorithm and a newly introduced Xpert-based algorithm within a routine operational setting. Other factors influencing the proportion of TB cases identified, including adherence to diagnostic algorithms and changes in the proportion of the population tested, were assessed.

This study was part of a PROVE IT (**P**olicy **R**elevant **O**utcomes from **V**alidating **E**vidence on **I**mpac**T**) evaluation (http://www.treattb.org/) to assess the impact of new molecular diagnostics on the diagnosis and treatment of tuberculosis.

## Methods

### Setting

The study was undertaken in Cape Town, South Africa, a city with a high TB burden with 28,658 cases registered in 2011. The TB case notification rate was 752/100,000 population. Amongst the 97% of TB cases tested for human immunodeficiency virus (HIV), 47% were co-infected. (Source: J. Caldwell, Routine TB Programme Data, Cape Town Health Directorate).

**Fig 1:**
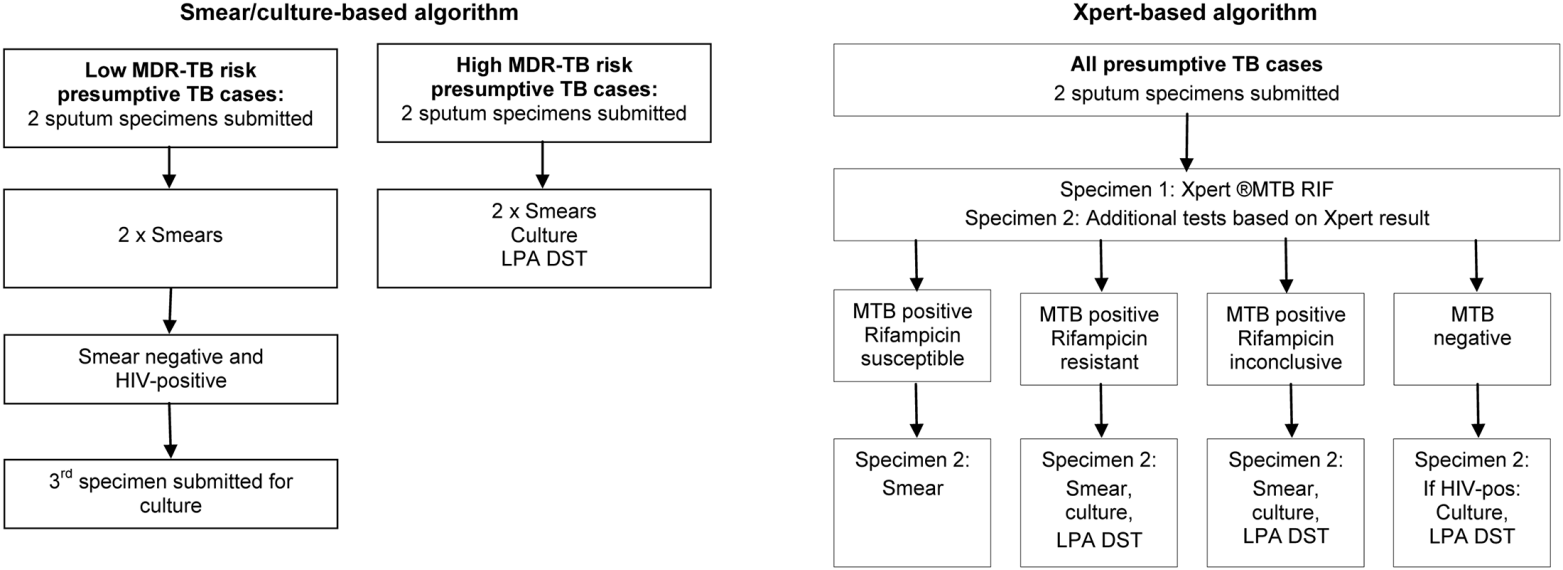
Testing protocols in the smear/culture and Xpert^®^ based TB diagnostic algorithms in PHC facilities in Cape Town. The diagram shows the simplified sequence of TB diagnostic tests recommended in each algorithm and the action taken based on test results. Low MDR-TB risk was defined as ≤four weeks previous TB treatment and high MDR-TB risk as >four weeks previous TB treatment, from congregate settings or with a known MDR-TB contact. Abbreviations: TB—tuberculosis, LPA—line probe assay, DST—drug susceptibility testing, HIV—human immunodeficiency virus, MTB—mycobacterium tuberculosis, PHC—primary health care.

Provincial and municipal health authorities provided free TB diagnostic services at 142 primary health care (PHC) facilities in eight sub-districts. Prior to August 2011, a smear/culture-based algorithm was used (Fig 1). Policy required all presumptive TB cases to be evaluated through two spot sputum specimens, taken 1-hour apart. Both specimens were chemically treated (using bleach or sodium hydroxide / n-acetyl cysteine), centrifuged, stained with auramine and examined by fluorescence microscopy. In high MDR-TB risk cases, the second specimen underwent liquid culture (BACTEC^™^ MGIT^™^ 960) and drug susceptibility testing (DST) using the GenoType^®^ MTBDRplus (Hain LifeScience GmbH, Nehren, Germany) line probe assay (LPA). Smear-negative, HIV-infected, low MDR-TB risk cases were required to submit a third specimen for culture.

From August 2011 to February 2013 an Xpert-based algorithm was phased into the eight health sub-districts with Xpert replacing smear microscopy (Fig 1). All presumptive TB cases submitted two sputum specimens. The first was tested with Xpert; if MTB was detected the second underwent smear microscopy to enable smear-conversion monitoring during treatment. In HIV-infected cases with negative Xpert tests, the second specimen underwent culture (in view of the lower Xpert-sensitivity in these cases [4]).

Samples were initially tested at a central national health laboratory. Three sub-district laboratories were established in 2013 to perform Xpert tests and smear microscopy. Results for all samples were entered into a networked electronic laboratory database.

#### Definitions

A *presumptive TB case* was defined as an individual with pre-treatment sputum samples submitted for diagnostic purposes and a *TB case* as an individual with one or more smears positive and / or culture positive for MTB and / or MTB detected on Xpert.

*Low MDR-TB risk* was defined as ≤four weeks previous TB treatment and *high MDR-TB risk* as >four weeks previous TB treatment, from congregate settings or with a known MDR-TB contact.

A *PHC site* consisted of municipal and provincial health facilities linked to their satellite and mobile facilities and to each other if within a single geographic location (to account for shared diagnostic services).

### Study design, population and timeframes

**Fig 2:**
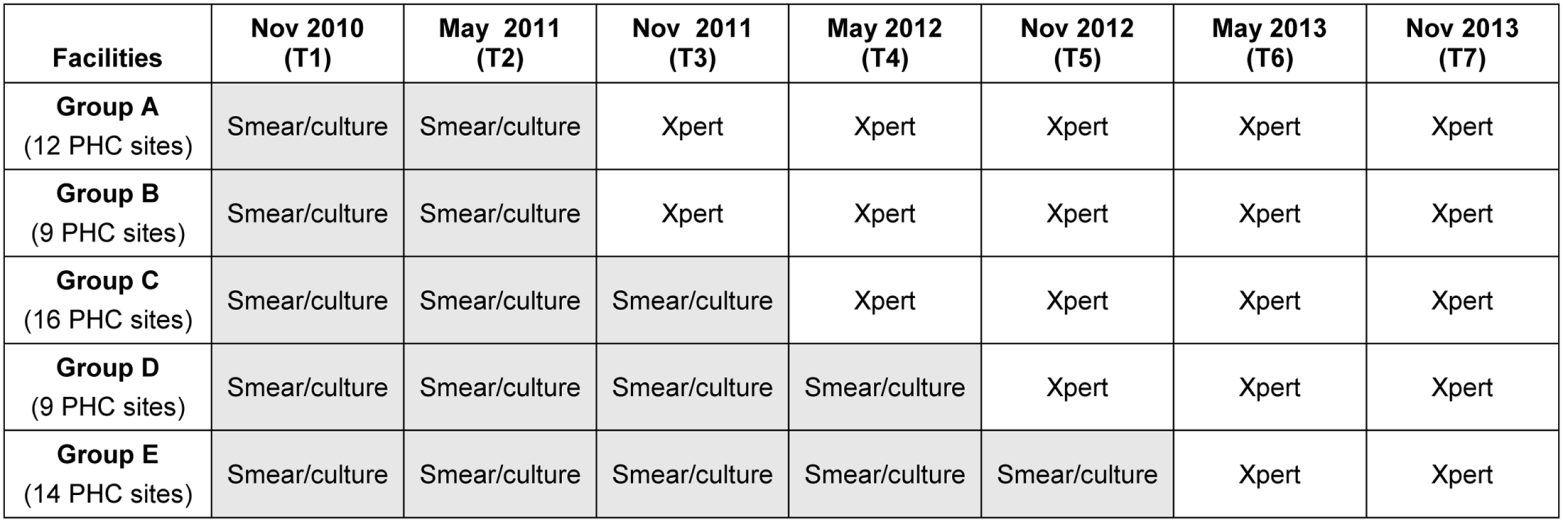
A non-randomised stepped-wedge evaluation of TB yield in five PHC groups as they transitioned from the smear/culture to the Xpert^®^ based algorithms in Cape Town. This figure shows the TB diagnostic algorithm in place in 5 groups of PHC sites over the seven time-points (T1 to T7) used in the analysis. All sites initially had a smear/culture-based algorithm in place. The Xpert-based algorithm was introduced in August 2011 in Group A, in October 2011 in Group B, in February 2012 in Group C, in October 2012 in Group D and in February 2013 in Group E. With the exception of one PHC site, the groups represent all the sites within a sub-district. Abbreviations: TB—tuberculosis, PHC—primary health care.

We used a non-randomised stepped-wedge study design to assess TB yield in five groups of PHC sites over seven one-month time-points (T1 to T7) as they changed from using the smear/culture-based algorithm to the Xpert-based algorithm (Fig 2).

We included 60 PHC sites (five of the eight sub-districts in Cape Town) that had implementation of either the smear/culture or the Xpert-based algorithm in October to December 2011. The three sub-districts transitioning in this quarter were excluded to avoid overlap between algorithms within the time-point. Other time-points were selected to also avoid overlap.

The study population included all presumptive TB cases with sputum test results recorded in the electronic laboratory database in Cape Town for the selected facilities and time periods.

### Data sources and management

The National Health Laboratory Services provided TB test data from the electronic laboratory database for 2010–2013. Data included patient demographic information, MDR-TB risk category, test type (smear, culture, or Xpert) and result.

Data for each time-point and the preceding and following months (i.e. a quarter’s data) were imported into MS-SQL. The full sequence of tests for individuals at T1 to T7 were derived by identifying all tests done in that month as well as in the preceding and following months using a combination of name, surname, clinic folder number and age or birth date.

### Analysis

TB diagnostic yield was calculated by dividing the number of TB cases identified (based on the full sequence of tests performed) by the total number of presumptive TB cases screened by algorithm and time-point.

Adherence to the smear/culture-based algorithm was assessed by calculating the proportion of cases with two smear tests and the proportion of smear-negative cases with culture tests. Adherence to the Xpert-based algorithm was assessed by calculating the proportion of cases with an Xpert test and the proportion of Xpert-negative cases with culture tests.

The proportion of presumptive TB cases tested in the five sub-districts was calculated by dividing the number of individuals tested in the quarter by the mid-year population estimates from Statistics South Africa [12].

Descriptive statistics by algorithm are presented using frequencies, means and standard deviation. We used a binomial regression model, adjusted for site-level clustering, to estimate the differences in TB yield between algorithms and assess temporal trends. This model was used to assess the effect of MDR-TB risk on TB yield and to evaluate trends in testing over time. All analyses were undertaken using STATA 12 (StataCorp).

### Ethics statement

The Health Research Ethics Committee at Stellenbosch University (IRB0005239) (N10/09/308) and Ethics Advisory Group at The International Union Against Tuberculosis and Lung Disease (59/10) approved the study. A waiver of informed consent was granted for use of routine data. The City Health Directorate, Western Cape Health Department and National Health Laboratory Service granted permission to use routine health data. Data sharing is possible following approval by the National Health Laboratory Service.

## Results

### Comparing the characteristics of presumptive TB cases evaluated by algorithm

The smear/culture-based algorithm included 24,000 presumptive TB cases and the Xpert-based algorithm 30,393 (Table 1). Age and gender distributions were similar. The smear/culture-based algorithm contained a larger proportion of high MDR-TB risk cases (26% compared to 24%, p<0.001).

**Table 1:** Comparing the characteristics of presumptive TB cases evaluated by algorithm.

	Smear/culture-based algorithm n = 24,000	Xpert-based algorithm n = 30,393	p-value
**Age (Years)**			
Mean	36	36	0.222
SD	15	15	
Range	2 to 98	1 to 100	
**Gender**			
Female (%)	11,402 (48)	14,310 (48)	0.983
Male (%)	11,788 (49)	14,789 (49)	
**Patient category**			
Low MDR-TB risk (%)	15,070 (63)	19,674 (65)	<0.001
High MDR-TB risk (%)	6,233 (26)	7,245 (24)	

Summary data are shown for presumptive TB cases evaluated in the existing smear/culture and newly introduced Xpert-based algorithm in primary health sites in Cape Town. Data are shown for recorded variables only. Missing data are included in the calculation of percentages. Low MDR-TB risk was defined as ≤four weeks previous TB treatment and high MDR-TB risk as >four weeks previous TB treatment, from congregate settings or with a known MDR-TB contact. Abbreviations: TB—tuberculosis, MDR-TB—multidrug-resistant tuberculosis, SD—standard deviation.

### Comparing TB yield by algorithm and trends over time

TB yield was 20.9% (95% CI 19.9% to 22.0%) in the smear/culture-based algorithm compared to 17.9% (95%CI 16.4% to 19.5%) in the Xpert-based algorithm with a risk difference of -3.0% (95% CI -4.3% to -1.7%) (p<0.001) (Table 2). Adjusting for the differences in MDR-TB risk produced similar TB yields: 20.1% in the smear/culture-based algorithm compared to 17.2% in the Xpert-based algorithm and a risk difference of -2.9% (p<0.001).

**Table 2:** A comparison of TB yield in smear/culture and Xpert^®^ based algorithms and trends over time.

	TB yield	95% CI	Risk difference (95% CI)	p-value
**All cases (simple model)**				
Smear/culture-based algorithm	20.9%	19.9% to 22.0%	-3.0% (-4.3% to -1.7%)	<0.001
Xpert-based algorithm	17.9%	16.4% to 19.5%		
**All cases adjusted for MDR-TB risk**				
Smear/culture-based algorithm	20.1%	18.8% to 21.5%	-2.9% (16.4% to 19.5%)	<0.001
Xpert-based algorithm	17.2%	15.4% to 19.0%		
**Amongst low MDR-TB risk cases (simple model)**				
Smear/culture-based algorithm	20.3%	19.0% to 21.5%	-3.2% (-4.9% to -1.4%)	<0.001
Xpert-based algorithm	17.1%	15.1% to 19.1%		
**Amongst high MDR-TB risk cases (simple model)**				
Smear/culture-based algorithm	24.4%	23.2% to 25.6%	-2.3% (-3.8% to -0.7%)	0.004
Xpert-based algorithm	22.1%	21.0% to 23.3%		
**For cases in both the smear/culture and Xpert-based algorithms by time point (simple model)**				
T1 (November 2010)	23.6%	22.2% to 25.1%	-0.9% (-1.2% to -0.6%)^a^	<0.001
T2 (May 2011)	20.4%	19.1% to 21.8%		
T3 (November 2011)	19.4%	18.3% to 20.5%		
T4 (May 2012)	17.4%	15.8% to 19.1%		
T5 (November 2012)	19.5%	17.8% to 21.2%		
T6 (May 2013)	16.6%	15.0% to 18.2%		
T7 (November 2013)	17.5%	15.0% to 20.0%		
**All cases adjusted for temporal trends**				
Smear/culture-based algorithm	19.1%	17.6% to 20.5%	0.3% (-1.8% to 2.3%)	0.796
Xpert-based algorithm	19.3%	17.7% to 20.9%		

The table shows TB yield comparisons between the smear/culture and Xpert-based algorithms and temporal trends in yield at time-points T1 to T7 in primary health sites in Cape Town. The outputs are from a binomial regression model, adjusted for clustering of presumptive TB cases at sites. In the simple model adjustments have not been made for differences in MDR-TB risk profiles between the algorithms or for temporal trends.

^a^This is the mean risk difference per time point and 95% confidence interval.

Abbreviations: TB—tuberculosis, MDR-TB—multidrug-resistant tuberculosis, CI—confidence interval.

Amongst *low MDR-TB risk cases*, TB yield in the smear/culture-based algorithm was 20.3% (95% CI 19.0% to 21.5%) compared to 17.1% (95% CI 15.1% to 19.1%) in the Xpert-based algorithm with a risk difference of -3.2% (95% CI -4.9% to -1.4%) (p<0.001). Amongst *high MDR-TB risk cases*, TB yield was 24.4% (95% CI 23.2% to 25.6%) and 22.1% (95%CI 21.0% to 23.3%) respectively with a risk difference of -2.3% (95% CI -3.8% to -0.7%) (p = 0.004).

**Fig 3:**
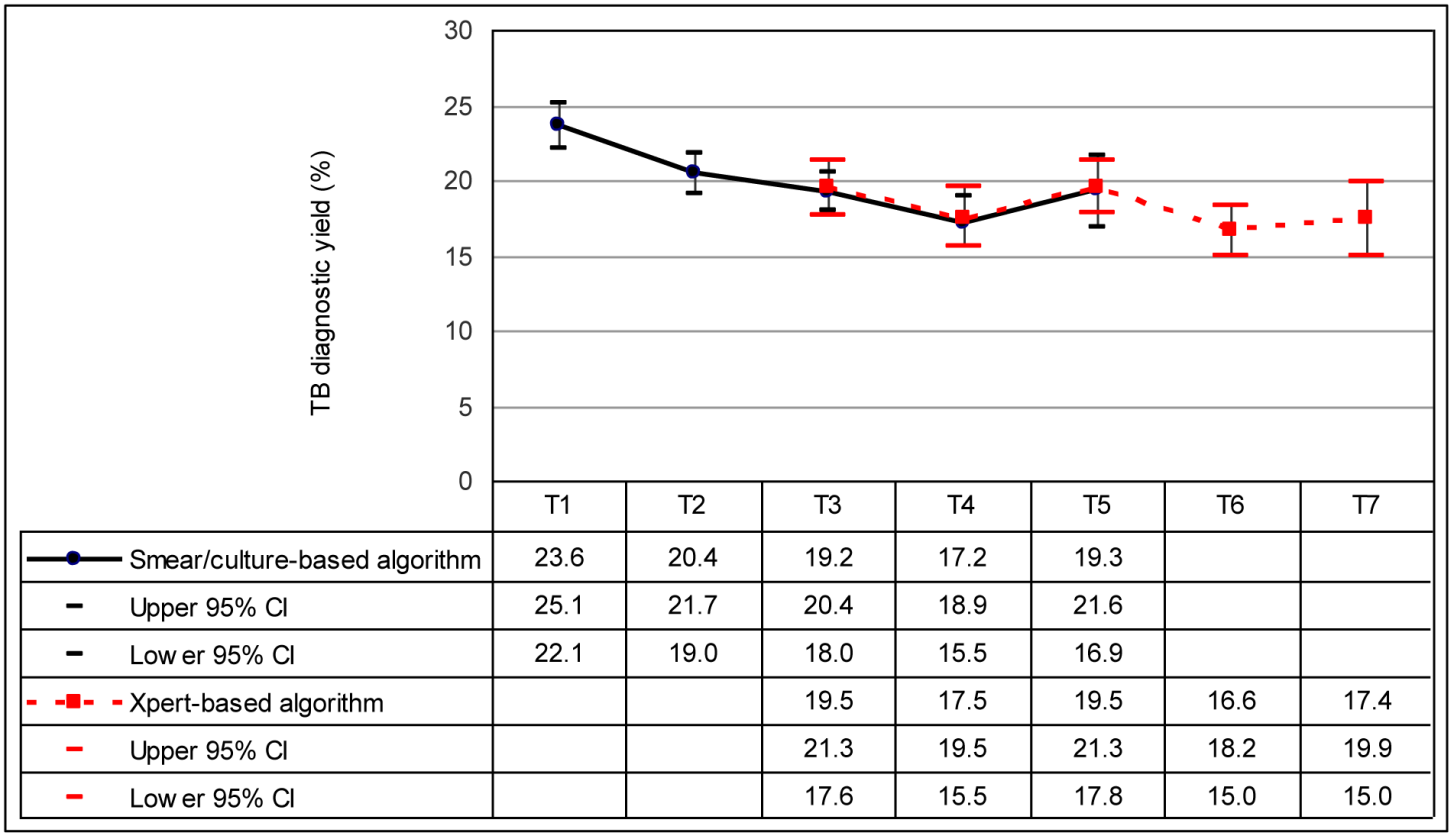
TB yield in the smear/culture and Xpert^®^ based algorithms at PHC sites in Cape Town by time-point. The graph shows the proportion of presumptive cases identified with TB (and 95% confidence intervals) in the smear/culture and Xpert-based algorithms as PHC sites changed from the former to the latter over seven time points (T1 to T7). Estimates derived from the binomial regression analysis were adjusted for clustering of cases at PHC sites. Time points were as follows: T1 = November 2010, T2 = May 2011. T3 = November 2011, T4 = May 2012, T5 = November 2012, T6 = May 2013, T7 = November 2013. Abbreviations: TB—tuberculosis, PHC—primary health care, CI—confidence interval.

There was a declining trend in TB yield in both the smear-culture and Xpert-based algorithms (Fig 3). Overall, TB yield was 23.6% (95% CI 22.2 to 25.1%) at T1 compared to17.5% (95% CI 15.0% to 20.0%) at T7 with a mean risk difference of -0.9% (95% CI -1.2% to -0.6%) (p<0.001) per time point (Table 2).

When estimates were adjusted for the temporal trend, there was no significant difference between the algorithms: the proportion of TB cases identified in the smear/culture based algorithm was 19.1% (95% CI 17.6% to 20.5%) compared to 19.3% (95% CI 17.7% to 20.9%) in the Xpert-based algorithm with a risk difference of 0.3% (95% CI -1.8% to 2.3%) (p = 0.796) (Table 2).

### Adherence to algorithms

Amongst *low MDR-TB risk* presumptive TB cases, 83.9% in the smear/culture-based algorithm had two smear tests and 35.5% of smear-negative cases had a culture test (Table 3). In the Xpert-based algorithm in comparison, 77.2% had an Xpert test and 17.9% of Xpert-negative cases had a culture test. The proportion of smear and Xpert-negative cases with culture tests could not be stratified by HIV-status as HIV data were unavailable for time-points T1 to T5. Amongst all low MDR-TB risk cases, the proportion with either two smear tests or an Xpert test increased from 84.6% at T1 to 94.3% at T7. The binomial regression analysis showed a mean increase in cases with either two smears or an Xpert test of 1.8% (95% CI 1.1% to 2.6%) (p<0.001) per time-point and a mean decrease of 3.7% (95% CI -4.8% to -2.6%) (p<0.001) in smear or xpert-negative cases with culture tests per time-point.

**Table 3:** Temporal trends in TB testing in the smear/culture and Xpert^®^ based algorithms.

			**Proportion of presumptive TB cases with tests done at each time point (%)**							
	**Algorithm**	**Tests**	**T1**	**T2**	**T3**	**T4**	**T5**	**T6**	**T7**	**All**
**For low MDR-TB risk cases**	Smear/culture-based	Two smears	84.6	84.7	86.0	89.5	59.9	-	-	83.9
		Smear-negative with culture	34.3	36.8	33.4	41.5	30.3	-	-	35.5
	Xpert-based	Xpert	-	-	64.3	75.6	68.3	82.3	84.9	77.2
		Xpert-negative with culture	-	-	26.6	16.6	18.7	18.3	15.5	17.9
	Overall	Two smears	84.6	84.7	61.6	38.8	32.2	12.5	9.3	45.4
		Xpert	-	-	25.3	52.1	61.2	82.3	84.9	44.7
		Smear-negative with culture	34.3	36.8	34.2	39.6	37.1	50.9	25.2	36.2
		Xpert-negative with culture	-	-	26.3	16.7	18.8	18.3	15.5	17.9
**For high MDR-TB risk cases**	Smear/culture-based	Two smears	81.9	81.7	82.7	84.4	67.9	-	-	81.5
		Smear-negative with culture	84.6	86.7	84.2	85.7	82.7	-	-	85.2
	Xpert-based	Xpert	-	-	53.0	56.0	56.7	71.3	78.0	64.6
		Xpert-negative with culture	-	-	44.8	35.4	39.4	42.9	40.2	40.6
	Overall	Two smears	81.9	81.7	56.8	47.4	38.6	22.5	15.1	51.2
		Xpert	-	-	25.7	38.6	50.6	71.3	78.0	35.3
		Smear-negative with culture	84.6	86.7	85.0	80.4	76.9	70.3	75.4	82.6
		Xpert-negative with culture			44.3	35.4	39.3	42.9	40.2	40.5
**All cases**	Smear/culture-based	Culture	51.9	52.9	48.1	58.3	40.9	-	-	51.5
	Xpert-based	Culture	-	-	42.9	34.2	36.8	33.5	24.5	32.8
	Overall	Culture	51.9	52.9	45.9	41.3	37.5	33.5	24.5	41.1
			**Trends in laboratory testing over time**							
			**T1**	**T2**	**T3**	**T4**	**T5**	**T6**	**T7**	**All**
**All cases**	Smear/culture-based	Presumptive TB cases screened	8,083	7,842	4,465	2,269	1,341	-	-	24,000
		TB cases diagnosed	1,911	1,601	804	427	276	-	-	5,019
	Xpert-based	Presumptive TB cases screened	-	-	3,309	5,371	5,873	7,714	8,126	30,393
		TB cases diagnosed	-	-	702	906	1,133	1,281	1,422	5,444
**Proportion of the population tested per quarter**			0.95	0.93	0.85	0.84	0.80	0.84	0.89	-

Data on the proportion of presumptive TB cases with appropriate tests undertaken is shown by MDR-TB risk profile, algorithm and time-point. The analysis includes the full sequence of tests undertaken for individuals identified at each time point, including tests in the preceding and following months. The proportion of the population tested per quarter was calculated based on the number of presumptive TB cases tested in the quarter divided by the mid-year population estimates for the five sub-districts included in the analysis. Abbreviations: TB—tuberculosis, MDR-TB–multidrug-resistant tuberculosis

Amongst *high MDR-TB risk* presumptive TB cases, 81.5% in the smear/culture-based algorithm had two smear tests and 85.2% of smear-negative cases had a culture test (Table 3). In the Xpert-based algorithm in comparison, 64.6% had an Xpert test and 40.6% of Xpert-negative cases had a culture test. Amongst all high MDR-TB risk cases, the proportion with either two smear tests or an Xpert test increased from 81.9% at T1 to 93.1% at T7. The binomial regression analysis showed a mean increase in cases with either two smears or an Xpert test of 2.3% (95% CI 1.7% to 3.0%) (p<0.001) per time point and a mean decrease of 8.0% (95% CI -9.0% to -7.0%) (p<0.001) in smear or Xpert-negative cases with culture tests per time point.

The proportion of presumptive TB cases with a culture test was higher in the smear/culture-based algorithm (51.5%) than in the Xpert-based algorithm (32.8%) (Table 3). The binomial regression analysis showed a mean decrease in cases with a culture test of 4.7% (95% CI -5.1% to -3.8%) (p<0.001) per time-point.

### Trends in the proportion of the population tested over time

The trend in the proportion of the population tested over time varied from a high of 0.95% at T1 to a low of 0.80% at T5 and up to 0.89% at T7 (Table 3). There was a small decline in the proportion of the population tested over time (slope -0.00007, p<0.001).

## Discussion

South Africa introduced Xpert as a replacement for smear microscopy for all presumptive TB cases in 2011. Since Xpert [4] has a higher sensitivity than smear microscopy [5,6,13] we expected to find a higher TB yield in the Xpert-based algorithm. The introduction of an Xpert-based algorithm however had no impact on TB yield in this routine operational setting.

A study in the rural Western Cape also found comparable TB yields amongst cases tested centrally through smear microscopy and Xpert [14]. Another study amongst HIV-infected cases enrolling for antiretroviral treatment in Zimbabwe found that the proportion of TB cases identified with Xpert was not significantly different to that from smears evaluated through fluorescence microscopy at a central laboratory [15]. The latter differs from a similar study in Cape Town that showed significant differences in the proportion of TB cases identified with Xpert and with smear microscopy amongst culture-confirmed cases enrolling for antiretroviral treatment [16].

Demonstration studies done on a moderate scale and at sites selected for good performance tend to over-estimate test efficacy [17] and may not reliably reflect findings in routine practice. Many of the studies reporting a significant increase in sensitivity for Xpert compared to smears used direct light microscopy for comparison in the majority or all or their sites [18–21]. Smear microscopy performance at our high throughput central laboratory may be better than that reported from peripheral microscopy units due to both greater proficiency and to technical aspects including chemical treatment, centrifugation and fluorescence microscopy, which have all been shown to increase yield [5,6,13]. The frequent use of culture tests in the smear/culture-based algorithm may also have contributed to the yield parity between algorithms and is discussed further below.

We found a decline in TB yield over time; the decline in the smear/culture-based algorithm was continued under the Xpert-based algorithm, with no change point observed. To assess whether adherence to algorithms may have contributed to this temporal trend, we assumed “diagnostic equipoise” i.e. that sensitivity was not lower with Xpert than with two smears. We assessed the proportion of low and high MDR-TB risk cases with either two smears or an Xpert test at each time point. Both showed increases over time, indicating that although adherence to the Xpert-based algorithm was sub-optimal, cases were better-off than previously for initial testing.

As HIV data for presumptive TB cases were not available for all time-points, we could not assess adherence to the second step of the algorithm (i.e. the proportion of HIV-infected, smear- or Xpert-negative cases with a culture test). Overall, we found a higher proportion of smear-negative cases compared to Xpert-negative cases with culture tests. Since a higher proportion of HIV-infected cases may initially have been diagnosed by Xpert than by smear, the extent to which this may reflect poorer adherence to the algorithm cannot be ascertained. It is possible that poor adherence to the Xpert-negative algorithm for HIV-infected cases contributes to the temporal trend to some extent.

The last few years has seen national efforts to intensify TB case-finding. As the proportion of the population tested increases, one would expect to find a decrease in TB yield. Our study found a minimal decline in the proportion of the population tested over time and this is therefore unlikely to have contributed to the temporal decline in TB yield.

We do not have local TB prevalence estimates. A Cape Town study reported a decrease in TB prevalence following antiretroviral treatment roll-out in one community [22]. It is possible that the rapid scale-up of antiretroviral treatment in South Africa may have contributed to a declining TB prevalence and to the temporal decline in TB yield in this study. This postulate is supported by a study reporting a decrease in the number of laboratory confirmed PTB cases in South Africa from 2011 to 2012 [23].

### Strengths and limitations

Diagnostic tests are rarely used in isolation. A unique aspect of this study is that we assessed TB diagnostic yield from each algorithm and not from individual tests. This reflected the variability that occurs in routine clinical practice, including both poor adherence to algorithms as well as the appropriate pursuit of a TB laboratory diagnosis after initial negative tests. Many presumptive TB cases are likely to undergo a sequence of tests and although this has been assessed in modelling studies [24,25], it is rarely reported from empirical field studies.

We used a stepped-wedge analysis, deemed appropriate where practical, financial or logistic constraints prevent the intervention being simultaneously introduced to all facilities [26]. An advantage of this method is that clusters act as their own controls and time-points prior and subsequent to the transition allow temporal trends to be assessed. In our study the introduction of the Xpert-based algorithm was determined by the health services based on operational requirements and not randomly assigned. Allocation bias may have influenced TB yield at time-points T3 to T5. The impact is difficult to quantify as the new algorithm was first introduced in areas with both high TB and HIV prevalence.

The extent to which results can be generalised is limited by the urban setting and good health and laboratory infrastructure, including access to liquid culture. Additional evidence is required from other settings particularly where culture is not routinely available.

### Implications for policy and practice

The failure to find an increase in TB notification rates following Xpert implementation has been attributed to high rates of empirical treatment [15,20,27]. The efficiency of the diagnostic algorithm in some settings may also contribute. Our data showed comparable TB diagnostic yields in the smear/culture and Xpert-based algorithms, perhaps partly attributable to the high proportion of cases with a culture test in the former.

Several studies have reported other benefits with Xpert, including early TB [21,28] and MDR-TB treatment initiation [29,30] and reduced MDR-TB patient costs [31]. However, the high health system costs of Xpert [3,32] and lack of impact on TB morbidity and/or mortality [15,21,33] are cause for concern. These factors together with the failure to find an increase in TB diagnostic yield suggests that Xpert may not have the anticipated impact on TB control and its role may need to be reviewed. Studies are required to assess whether Xpert can be used more cost-effectively, for example as a rapid screening test for rifampicin resistance in smear or culture-positive cases.

## Conclusion

Despite South Africa making great strides in expanding diagnostic services, including access to rapid tests through nation-wide implementation of Xpert, the proportion of TB cases diagnosed through laboratory tests in Cape Town has not increased. The historic smear/culture-based algorithm was as effective in identifying TB cases as the newly introduced Xpert-based algorithm. Additional studies are required to assess whether improving adherence to algorithms, particularly for Xpert-negative, HIV-infected individuals, will increase TB yield. Ultimately, difficult questions may need to be asked and answered about the future use of Xpert in resource-constrained settings where effective smear and culture-based algorithms are in place.
